# The role of paternal and maternal warmth and hostility on daughter’s psychosocial outcomes: The insidious effects of father warmth combined with high paternal hostility

**DOI:** 10.3389/fpsyg.2023.930371

**Published:** 2023-03-20

**Authors:** Julia Dmitrieva, Emma V. Espel

**Affiliations:** ^1^Department of Psychology, University of Denver, Denver, CO, United States; ^2^RMC Research Corporation, Denver, CO, United States

**Keywords:** father-daughter relationship, romantic relationship, internalizing, parental warmth, parental hostility, education and career goals

## Abstract

**Introduction:**

Despite the well-accepted view on the importance of parental warmth and parental hostility for adolescent development, few studies have examined the joint interactive effects of these two key aspects of parenting. Furthermore, research comparing maternal and paternal parenting is limited, with the father-daughter relationship during adolescence remaining one of the more understudied familial contexts. Given that family processes are key for the intergenerational transmission of inequality, these parent–child relationships may be especially important for youth at risk for exposure to violence.

**Objectives:**

Using a sample of juvenile female offenders, this study examined the associations between the perceived warmth and hostility in the father-daughter and mother-daughter relationships on daughters’ depressive symptoms, anxiety symptoms, romantic partner warmth, romantic partner hostility, and the daughter’s sense of agency. We hypothesized that high perceived parental warmth would moderate the effects of parental hostility by protecting daughters from the negative effects of parental hostility, with stronger effects for the father-daughter than the mother-daughter relationship.

**Results:**

In contrast, our paternal relationship findings across four of the five outcomes suggest a moderation in the opposite direction – that is, high perceived father warmth exacerbates the deleterious effects of father hostility on daughters’ depressive symptoms, anxiety, romantic partner warmth, and romantic partner hostility. Maternal warmth, and not hostility, had a direct association with these four outcomes, with stronger explanatory power shown for the father-daughter than the mother-daughter model. Higher agency was associated with maternal hostility only.

**Conclusion:**

Our findings suggest that daughters might be modeling and internalizing the relationship with their fathers (for better or worse) when they perceive it as warm and supportive. Consequently, adolescent girls whose fathers exhibit hostile behavior may benefit from emotional distancing from their fathers.

## 1. Introduction

The parent–child relationship is one of the most critical contexts affecting youth developmental outcomes, such as internalizing, peer and romantic relationship quality, and achievement outcomes. Whereas positive aspects of the parent–child relationship, such as closeness, support, and communication, are associated with better psychosocial outcomes, negative aspects, such as conflict and harsh discipline, contribute to the development and maintenance of a range of psychosocial problems (Vazsonyi et al., 2003; Carlson, 2006). Whereas a number of studies have examined the role of parental warmth and parental hostility, as one of the key aspects of parenting, less is known about the interactive effect of these two parenting dimensions. As well, few studies differentiate among the unique influences of maternal and paternal behavior. The current study aims to explore whether parental warmth moderates the effects of parental hostility on daughter psychosocial outcomes, while differentiating the maternal and paternal effects, in a sample of heterosexual cisgender parents and their cisgender daughters. Our sample was recruited as part of the study of delinquent girls and thus represents a population at risk for both negative developmental outcomes and negative family dynamics.

Parents and the quality of the parent–child relationship serve a uniquely important role, with parents typically serving as major attachment figures and providing a foundation for early development of emotional and psychological health (Bowlby, 1980). Early attachment relationships serve as a framework within which children and adolescents develop their sense of self and relationship with important others (Sroufe, 2002; Thompson, 2006). These influences persist into middle childhood and adulthood, predicting emotion regulation (Sroufe, 2005), peer relationship quality and social competence (Schneider et al., 2001), school competence (Aviezer et al., 2002), and depressive symptomatology (Duggal et al., 2001; Milan et al., 2013). Attachment theory posits that the quality of the parent–child relationships serves as a model for future relationships, including romantic relationships, by shaping expectations and understandings of intimacy (Bowlby, 1980; Hazan and Shaver, 1987; Ainsworth, 1989). Indeed, the quality of the parent–child relationship can serve an important function in shaping adolescents’ relationship expectations and influencing their behaviors and experiences in their romantic relationships (Crockett and Randall, 2006; Simpson et al., 2007). From the social learning perspective, individuals learn how to behave in intimate relationships through witnessing behavior modeled for them in other close relationships (Bandura, 1973). In general, children of warm and supportive parents tend to have similarly warm and supportive romantic relationships (Connolly and Johnson, 1996; Furman et al., 2002). Conversely, negative interactions (i.e., conflict and annoyance) within adolescent romantic relationships are correlated with negative interactions within parent–child relationships (Furman et al., 2002).

### 1.1. Parental warmth and hostility

Research on the quality of parent–child relationships and their effects on youth psychosocial outcomes is deeply rooted in Baumrind (1966) work on parenting styles, demonstrating that authoritative parenting style – a style that is characterized by both high demandingness/control and warmth/responsiveness is associated with a wide variety of positive psychosocial outcomes (Larzelere et al., 2013). In contrast, authoritarian style (a harsh parenting style that is characterized by high control/demandingness and low warmth/responsiveness) and neglectful style (characterized by low control/demandingness and low warmth/responsiveness) are associated with higher internalizing symptoms (Yap and Jorm, 2015; Pinquart, 2017; Liu and Merritt, 2018).

Much of the research on parenting has continued to adopt the dimensional perspective that evaluates parents in terms of the degree of warmth and control, while further elaborating on different parenting behaviors that constitute these two dimensions. The warmth dimension generally represents parents’ acceptance, affection, sensitivity, and involvement (Rohner et al., 2005; Pomerantz and Thompson, 2008). More specifically, the affection and involvement aspects of warmth have been linked with youth mental health outcomes, social competence, and academic achievement (Wood et al., 2003; Dmitrieva et al., 2004; Kim et al., 2007; McLeod et al., 2007a, 2007b; Chung et al., 2008; Yagmurlu and Sanson, 2009; Chang et al., 2010; Yap et al., 2014; Yap and Jorm, 2015). However, hyper-involvement in the form of “helicopter parenting” or controlling (as opposed to autonomy-supporting) has been linked to lower psychological and achievement outcomes (Pomerantz et al., 2007; LeMoyne and Buchanan, 2011; Schiffrin et al., 2013).

The control dimension ranges from supervision to psychological power assertion and harsh discipline. Research differentiating the tactics that constitute control, demonstrates positive psychological and achievement outcomes in association with beneficial aspects of control such as parental monitoring and supervision (Barber et al., 1994; Pettit et al., 2007; Akcinar and Baydar, 2014) and deleterious outcomes in association with harsh verbal and physical punishment (Fulton and Turner, 2008; Pinquart, 2017; Gorostiaga et al., 2019) and psychological control (i.e., power assertion, intrusiveness, and withdrawal of affection; Bean et al., 2003; Aunola and Nurmi, 2005; El-Sheikh et al., 2010). As one of the extreme ends of negative parental control, parental hostility is associated with youth internalizing (Allen et al., 1998; Muris et al., 2004), and behavioral problems (Backman et al., 2021). The role of parental hostility has been especially central in the studies of intergenerational transmission of violence, showing that exposure to parental violence is a major factor increasing the risk of dating violence (Foshee et al., 1999; Lewis and Fremouw, 2001; Hendy et al., 2003; Kwong et al., 2003; Simons et al., 2008).

Although the literature on parental warmth and control dimensions conceptualizes hostile parental control as the control dimension that is distinct from parental warmth, others have discussed parental hostility as a construct on the opposite end from nurturing and warmth along the single warmth-rejection dimension. This operationalization stems from Schaefer’s (1959) work identifying the warmth–hostility parenting dimension, based on ratings of high affection and parental sensitivity on one end, and rejection and punitiveness on the other. Research stemming from the PARTheory (Rohner, 2004; Rohner et al., 2005) continued to examine the acceptance-rejection continuum, where rejection can be expressed through physical, psychological, and verbal hostility (along with neglect) and is on the opposite end of the warmth dimension from nurturance and acceptance. This theory has generated wide empirical support, with parental acceptance-rejection being linked to adolescent depression and youth prosocial behavior across many cultural groups (Rohner and Britner, 2002; Bradford et al., 2003; Gülay, 2011).

Recent research calls for a more multidimensional approach to constructs related to parental warmth and hostility (Bornstein, 2015; Rious et al., 2019). Indeed, research with Chinese American, Mexican American, and Korean families shows that parents may simultaneously be characterized as both “warm” and “hostile” (Rohner and Pettengill, 1985; Chao, 1994; Garcia Coll and Pachter, 2002; Skinner et al., 2005; Chao and Otsuki-Clutter, 2011). Whereas few studies compare the effects of parental warmth and hostility on youth outcomes in a single model, emerging findings further support the distinction between parental warmth and hostility constructs by showing divergent outcomes in association with these variables. For example, Connor and Rueter (2006) study of 451 adolescents in Iowa reports that parental warmth, but not parental hostility, is associated with adolescent emotional distress (assessed with depressed mood, anxiety, and hostility). Similarly, Vaughan et al. (2021) show that parental warmth, and not hostility, is associated with youth prosocial behavior. In contrast, maternal hostility was associated with youth delinquency and aggression.

Although parenting constructs do not exert their influence alone but interact in a transactional system (Baumrind, 1991; Darling and Steinberg, 1993; Power, 2013), little is known about the potential interactive effect of parental warmth and hostility. In a rare exception, Deater-Deckard and Dodge (1997) tested parental warmth as a moderator of the concurrent effects of harsh discipline on child aggression from kindergarten through sixth grade. There was no association between harsh discipline and child aggression for families characterized by high levels of parental warmth, in contrast to low-warmth families, where this association was significant for all ages. McKee et al. (2007) found that high parental warmth buffers children from the negative effects of corporal punishment on behavioral problems, and McLoyd and Smith (2002) found that corporal punishment predicts increases in youth internalizing and externalizing problems in the context of low maternal warmth, but not in the context of high maternal warmth. In contrast, parental warmth was not found to moderate the effect of corporal punishment on externalizing problems among young children in China (Xing and Wang, 2017). Similarly, Lansford et al. (2014) found that parental warmth moderated the effect of corporal punishment on anxiety across eight countries. Surprisingly, the deleterious effect of corporal punishment on anxiety was the highest for children whose mothers were rated as warm in China, Jordan, Kenya, the Philippines, Thailand and the US. In a different pattern of results, high maternal warmth protected children from the negative effects of corporal punishment on anxiety for children in Columbia and Italy. Additional evidence of the interactive effects of parental warmth and hostility come from studies that employ the person-oriented approach to parenting. As such, Zheng et al. (2017) identified subgroups of parents based on trajectories of parental warmth and harsh discipline. Their findings show the highest risk for externalizing problems among children whose parents exhibit a pattern of high chronic harsh discipline and low/increasing levels of parental warmth. Thus, extant research provides some evidence that parental warmth moderates the effects of parental hostility on youth outcomes. However, the extent and direction of this effect may vary as a function of a sample and its sociocultural context.

### 1.2. The role of paternal and maternal warmth and hostility

Historically, parenting studies have placed greater emphasis on maternal parenting behaviors and/or relied on maternal reports of parenting as a whole. This emphasis on mothers stems from the erroneous and sexist assumptions that fathers are less consequential, whereas mothers are responsible for negative child outcomes (Phares et al., 2002; Cassano et al., 2006). Furthermore, the bulk of parenting research has focused on heterosexual cisgender parents and cisgender children. Given the cisgender heterosexual sample of the current study, our review focuses on gender differences among these groups, while acknowledging the limited nature of the literature reviewed in this section. The bias toward the study of the mother–child relationship is not wholly unwarranted given that, on average, mothers are responsible for more duties involving childcare and report being more involved and spending more time with adolescent children than fathers (Williams and Kelly, 2005; Allgood et al., 2012). Furthermore, mothers and fathers often exhibit similarities in their parenting behavior. For example, high levels of maternal support are often correlated with high levels of paternal support (Laible and Carlo, 2004; Videon, 2005; Goncy and van Dulmen, 2010). Yet, as social roles shift, the field’s traditional approach to studying parenting as a whole or with a mother-only bias may overlook important unique contributions of paternal parenting.

The past several decades have seen a continued transformation of traditional parenting roles. Conventional male responsibilities, such as working outside the home and supporting the family economically, shifted from the father alone to both the father and the mother, with domestic and child-caring responsibilities mirroring this shift (Cabrera et al., 2000). Although research has noted these changes, it has more often explored the effect of the mother’s engagement in the workplace (Videon, 2005), effects of father absence (Pleck, 1997) or predictors of father involvement with the child (Coley and Hernandez, 2006). Studies that do investigate father-child relationship quality demonstrate that maternal and paternal involvement each have independent effects on child and adolescent outcomes, including educational attainment, internalizing behavior, and behavioral outcomes (Cabrera et al., 2000; Flouri and Buchanan, 2004; Rinaldi and Howe, 2012; Suizzo et al., 2017). Furthermore, emerging research indicates that mothers and fathers may have distinct contributions to youth outcomes. For example, Day and Padilla-Walker (2009) found that fathers’ behavior (involvement and connectedness) may serve as a better predictor of adolescent externalizing and internalizing problems, whereas a mother’s behavior may have a unique influence on adolescent prosocial behavior and sense of hope. In other studies, paternal warmth has been shown to be a stronger correlate of adolescent emotional distress, as compared to maternal warmth (Connor and Rueter, 2006); whereas, maternal hostility (and not paternal hostility) has been linked to daughters receiving and sons both receiving and inflicting violence in a romantic relationship (Hendy et al., 2003). Khaleque and Rohner (2012) review of cross-cultural research on parental acceptance shows that, compared to maternal acceptance, paternal acceptance is at times a better predictor of psychological and behavioral adjustment. In contrast, a review of cross-cultural research on parental hostility (Khaleque, 2017) showed that, compared to paternal hostility, maternal hostility has a stronger effect on youth adjustment. Yet, another cross-cultural review found no differences between the effects of maternal and paternal warmth on youth psychological adjustment (Khaleque, 2013). The magnitude of the effects of maternal and paternal parenting behaviors may also change across the lifespan. As such, Connell and Goodman (2002) found that compared to fathers, mothers have a stronger effect on internalizing during early and middle childhood, whereas fathers have a stronger effect than mothers during adolescence.

There has been some theoretical work explaining why mothers and fathers may have divergent effects on their children. Attachment researchers have proposed that fathers-child attachment has a function of an “activation relationship” (Dumont and Paquette, 2013). Whereas mothers are seen to provide care and security, fathers could be more instrumental in satisfying the child’s need for stimulation and exploration. Other researchers have proposed that distinct maternal and paternal outcomes can be linked to family distribution of interpersonal power. As such, Rohner and Carrasco (2014) show that the effect of parental acceptance on their child’s psychological adjustment is stronger for the parent that is perceived by the child to hold greater interpersonal power and prestige. Machado et al. (2014) found that fathers’ perceived interpersonal power intensified the effect of paternal acceptance on daughters’ adjustment, whereas fathers’ perceived prestige intensified the effect of paternal acceptance on sons’ adjustment.

The father-daughter relationship is comparatively the most understudied of the parent–child dyads, as conventional reasoning assigns greater importance to the father-son relationship (Allgood et al., 2012). However, research shows that both sons’ and daughters’ psychological outcomes are associated with the parenting they receive from their fathers (Wenk et al., 1994; Van Wel et al., 2000; Palkovitz, 2002). A number of studies suggest that the father-daughter relationship is not only important but may have stronger implications in some domains, as compared to other parent-youth dyadic relationships. For example, results of a British study of 13,000 youth suggest greater childhood-through-adulthood stability in the father-daughter relationship closeness, as compared to the father-son relationship closeness (Flouri, 2005). Furthermore, compared to sons, daughters experienced a greater effect of their closeness with the father on the adult daughter’s depressive symptoms, own marriage, and educational and career success. In contrast, closeness with the mother predicted marital satisfaction, but not the psychological and academic outcomes. Stolz et al. (2005) found a cross-gendered effect of maternal and paternal support on adolescent depressed mood, such that paternal behaviors exert a stronger influence on daughters, whereas maternal behaviors exert a stronger influence on sons. In Sultana and Khaleque (2016) study, both maternal and paternal acceptance made independent contributions to adult sons’ adjustment, whereas only paternal acceptance had an independent effect on adult daughters’ adjustment.

Fathers may play an especially crucial and unique role in the development of daughters’ romantic relationships. The quality of the parent–child relationship can serve an important function in shaping adolescents’ relationship expectations and influencing their behaviors and experiences in their romantic relationships (Crockett and Randall, 2006; Simpson et al., 2007) and this transition from a parent as an attachment figure to a romantic partner begins in late adolescence (Furman and Wehner, 1994). The father-daughter relationship may serve a unique role of modeling other intimate opposite-sex relationships.

Indeed, a positive father-daughter relationship has been shown to be associated with better romantic relationship quality (Black and Schutte, 2006; Scharf and Mayseless, 2008; Last, 2009). Furthermore, the quality of the father-daughter relationship is more strongly associated with the romantic relationship quality than the quality of the mother-daughter relationship (Danes et al., 2006; Scharf and Mayseless, 2008). Similarly, daughters reporting a more negative relationship with their fathers (but not mothers) are more likely to have boyfriends who encourage antisocial behavior (Cauffman et al., 2008). Thus, an adolescent girl’s selection of a partner, as well as her behavior and expectations for romantic relationships, may be influenced by the quality of her relationship with her father. Overall, features of the father-daughter relationship, especially indicators of relationship quality such as warmth and hostility, appear to influence girls’ experiences in their romantic relationships. Taken together, past research suggests that fathers are an understudied but substantial source of influence on adolescent outcomes, perhaps especially for daughters.

### 1.3. The present study

**Figure 1:**
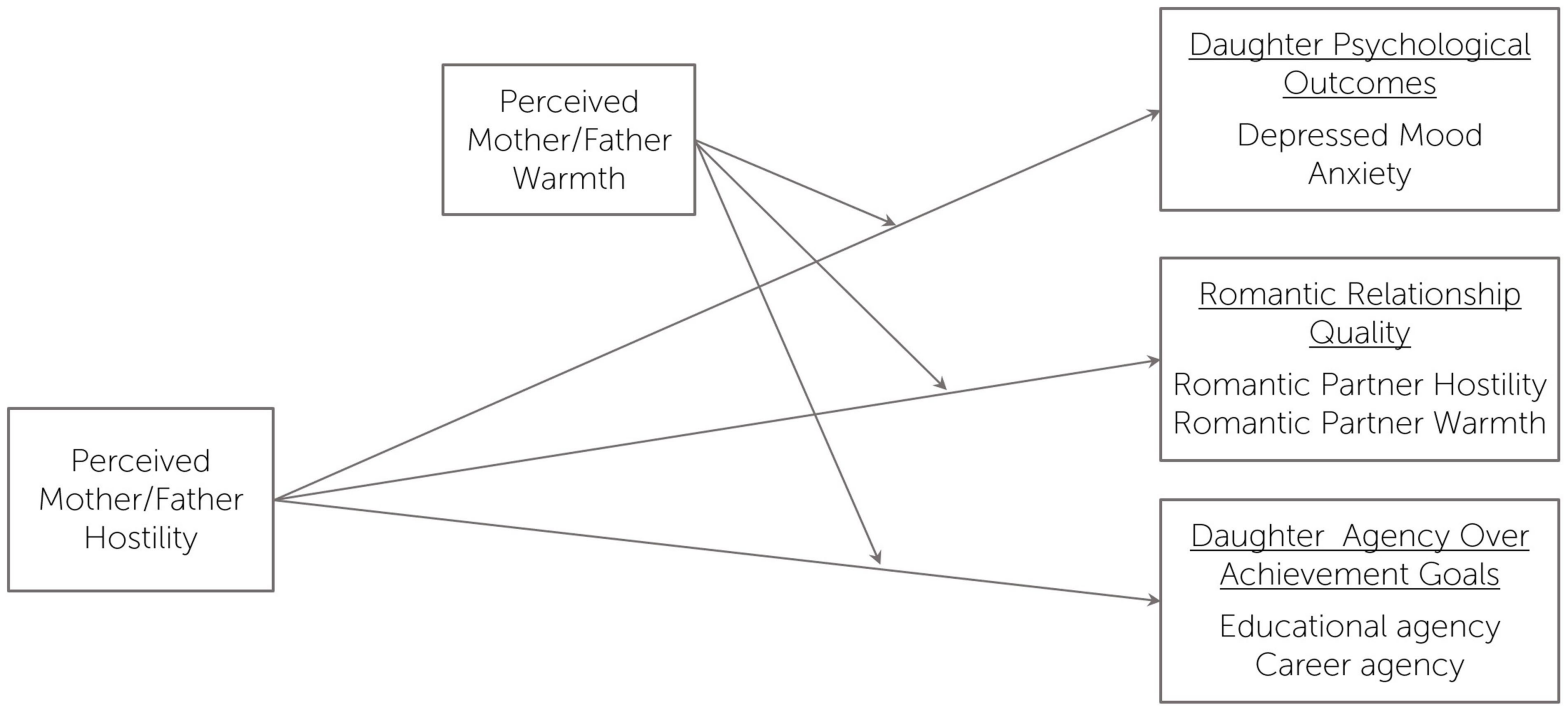
The hypothesized model.

The present study examined the combined effect of maternal and paternal warmth and hostility, as perceived by the daughters, on the daughter’s romantic relationship quality, depressive and anxiety symptoms, and career and educational goals in a sample of adolescent delinquent girls at risk for family dysfunction and maltreatment. In the last decade, there has been increased attention directed at improving our understanding of how social disadvantages are transmitted across generations. These key mechanisms of transmission often involve family dynamics and parenting (Lareau, 2000; McLanahan, 2004; Conger et al., 2010; Ermisch et al., 2012; Kalil, 2015). Whereas the literature on the effects of parenting among juvenile offenders has predominantly focused on delinquent boys, the current study aims to fill the gap in our understanding of parenting influences among delinquent girls. Importantly, the current study aims to identify the unique effects of parental warmth, hostility, and their interaction. Based on the emerging literature, we expect that parental warmth will moderate the effects of parental hostility on daughters’ psychosocial outcomes (see Figure 1), such that the co-occurrence of high parental hostility and low perceived parental warmth would result in a synergistic effect associated with the most pronounced negative outcomes, and high warmth will protect daughters from the negative effects of high hostility. In view of the gender differences in the effects of parental warmth and hostility, we expect stronger effects of fathers than mothers, especially for the daughter romantic relationship quality. Few studies have compared maternal and paternal warmth as a moderator of hostility, and none tested these comparisons across the range of internalizing, romantic relationship, and achievement outcomes included in this study. To our knowledge, this study is also the first to test warmth and hostility in mother-daughter and father-daughter relationships in a sample of delinquent girls.

## 2. Materials and methods

### 2.1. Participants

Participants took part in a study of adjudicated adolescent females in Denver, Colorado, between 2011 and 2014. All 90 participants were cis-gender females from ages 13–21 (M = 17.61, SD = 1.90) who had been adjudicated of a crime, and either were confined in a secure juvenile facility or served a probationary sentence. Out of the 90 participants, 61 reported being in a current romantic relationship at the time of the study. Those not in a relationship reported on their most recent relationship. Of these relationships, 83% were heterosexual and 17% were same-sex relationships with a cis-gender female. We examined participant sexual orientation as a potential covariate; however, it was not associated with relationship warmth or hostility (across parenting and romantic relationships). The sample was racially/ethnically diverse, with 39% identifying as non-Hispanic White, 29% identifying as Latina, 20% identifying as Black, and 11% identifying as other or bi-racial. Participants came from disadvantaged backgrounds, with average maternal and paternal education ranging between 11th grade and a high school diploma. We tested age, parental education, and race as potential covariates. Only age was significant in our multivariate models, and was therefore retained as a covariate in the final models.

### 2.2. Procedure

Interviews lasting approximately 2 h included questions involving participants’ experiences, feelings, romantic relationships, family relationships, and delinquent behavior. Participants were interviewed by undergraduate or graduate-level research assistants trained in data collection procedures either in person at the incarceration facility or, for those participants who were serving a probationary sentence, in a public place (e.g., a mall). Participant recruitment and procedures were approved by the university Institutional Review Board and participant reports were protected by the NIH Certificate of Confidentiality. All interviews were voluntary and confidential, and participants could refuse to answer questions with which they did not feel comfortable answering. Participants were compensated $30 for the interview.

### 2.3. Measures

**Perceived Warmth**. The same perceived warmth scale was used to assess daughter perceptions of maternal, paternal, and romantic partner warmth (Chen et al., 1998). The scale included seven questions, such as “He/she really understands me”; “I know that he/she will be there for me if I need him/her”; and “I do not feel that he/she really cares about me – reverse coded.” These questions were answered on a 6-point Likert scale (1 = Strongly Disagree to 6 = Strongly Agree). The scale had good internal consistency for fathers (α = 0.88) and mothers (α = 0.89), and adequate internal consistency for romantic partners (α = 0.70).

**Perceived Hostility**. Relationship hostility in parent-daughter and romantic partner relationships were measured using items from the Quality of Parental Relationships Inventory (Conger et al., 1994). The scale included 12 items, such as “When you and your mother/father/partner have spent time talking or doing things together, how often did he/she…Swear at you? Shout or yell at you because they were mad at you? Slap or hit you with their hands? Push, grab, hit or shove you?” These questions were answered using a 4-point Likert scale (0 = Never to 3 = Always). The scale had excellent internal consistency for fathers (α = 0.92), mothers (α = 0.93), and romantic partners (α = 0.91).

**Depressive Symptoms**. Adolescent depressive symptoms were measured with the 20-item Center for Epidemiologic Studies Depression Scale (CES-D; Radloff, 1977). Adolescents reported the frequency of depressive symptoms over the past month with values ranging from 1 (never) to 4 (almost every day). The scale had adequate internal consistency, α = 0.70.

**Anxiety Symptoms**. Anxiety symptoms were assessed using the 28-items from the Revised Children’s Manifest Anxiety Scale (RCMAS; Reynolds and Richmond, 1979). The scale includes a checklist of symptoms that include physiological symptoms, worry, high interpersonal sensitivity, and concentration. Adolescents reported whether those symptoms were present or absent for them, with responses summed up for a summary score. The scale had good internal consistency, α = 87.

**Agency over Career and Educational Goals**. Participants reported their immediate (1 year after their release) and long-term (10 years after their release) career and educational goals. They were next asked to report on the amount of control they felt they had over attaining these goals, using response categories that ranged from 0 = no control to 4 = complete control. The resulting career and educational agency scores were highly correlated (*r* = 0.75, *p* < 0.05) and were therefore combined into a single agency score. The total agency scale had a good internal consistency, α = 0.83.

## 3. Results

### 3.1. Descriptive statistics and bivariate correlations

As shown in Table 1, daughters on average reported higher levels of perceived romantic partner warmth than perceived father or mother warmth (t[60] = 4.31, *p* < 0.001 for the romantic partner comparison to the father and t[60] = 5.42, p < 0.001 for romantic partner comparison to the mother), whereas the differences between perceived maternal and paternal warmth were not significant (t[84] = 0.64, n.s.). Participants reported the highest levels of hostility in their relationships with their mothers, followed by their relationships with their fathers (t[84] = 2.78, *p* < 0.01 for maternal hostility vs. paternal hostility). Hostility in the romantic partner relationship was the lowest (t[60] = 2.96, *p* < 0.01 for the romantic partner vs. father comparison). Bivariate correlations were in the expected direction, with warmth in all three relationships being associated with lower depressive symptoms, maternal warmth being associated with lower anxiety, and romantic partner warmth being associated with higher agency. Perceived father hostility was not directly associated with any of the outcomes, whereas maternal and romantic partner hostility were associated with higher depressed mood.

**Table 1:** Zero-order correlations and descriptive statistics for the key study variables.

	(1)	(2)	(3)	(4)	(5)	(6)	(7)	(8)	(9)	(10)	(11)	M	(SD)
(1) Age	--												
(2) Parental education	−0.09	--											
(3) Perceived F warmth	−0.16	−0.09	--									4.52	(1.13)
(4) Perceived F hostility	−0.01	0.11	−0.50***	--								1.61	(0.54)
(5) Perceived M warmth	0.03	−0.11	0.33**	−0.22	--							4.40	(1.23)
(6) Perceived M hostility	0.03	0.19	−0.14	0.29**	−0.64***	--						1.88	(0.63)
(7) Perceived RP warmth	−0.20	0.20	0.40**	−0.26	0.22	0.04	--					5.39	(0.49)
(8) Perceived RP hostility	0.17	−0.20	−0.15	0.29	−0.25	0.17	−0.34*	--				1.35	(0.43)
(9) Depressive symptoms	−0.01	0.03	−0.33**	0.21	−0.35**	0.28**	−0.31*	0.41**	--			2.24	(0.55)
(10) Anxiety symptoms	0.14	−0.09	−0.15	0.08	−0.23*	0.11	−0.20	0.23	0.70***	--		13.39	(6.24)
(11) Agency	0.10	0.15	−0.02	0.03	0.04	−0.13	0.52***	−0.29	−0.16	−0.01	--	3.20	(0.78)

F = father, M = mother, RP = romantic partner.

* *p* < 0.05;

** *p* < 0.01;

*** *p* < 0.001.

### 3.2. The hypothesized model

We next tested the hypothesized models (Figure 1) for father-daughter and mother-daughter relationships, using Mplus 8.4 (Muthén and Muthén, 1998-2022). The models examined the interaction between the perceived parental warmth and hostility dimensions in their effects on depressive symptoms, anxiety symptoms, romantic partner hostility, romantic partner warmth, and agency, after controlling for age. First, each model (for father and mother) was tested separately, after which we trimmed paths that were not significant for either father or mother model. Thus, the paths from age to depressive symptoms, anxiety symptoms, and agency, as well as the interaction effect of parental warmth and parental hostility on agency were removed from the models. Next, the model coefficients for the father and mother models were compared using the multigroup approach, where path coefficients were constrained to be equal across the father-daughter and mother-daughter models and tested with the delta chi-square test. Significant interactions and regions of significance were probed using the model constraint and loop plots with 2,000 bootstrap draws. The use of model constraint command is used to create and test new parameters, in this case – the slopes of parental hostility at different levels of parental warmth. The loop option was used to plot the average effect and its 95% CI for the effects of parental hostility across the range of values of parental warmth. That is, the 95% CI fully above zero indicates a region of parental warmth values for which parental hostility has a significant positive effect. Conversely, the 95% CI fully below zero indicates a region of parental warmth values for which parental hostility has a significant negative effect.

### 3.3. The father-daughter relationship model

**Figure 2:**
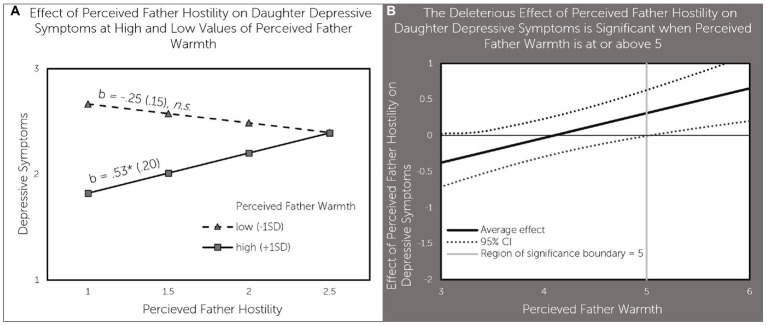
Perceived father warmth moderates the effect of perceived father hostility on daughter depressive sympotoms **(A)** effect of perceived father hostility on daughter depressive symptoms at high and low values of perceived father warmth; **(B)** the deleterious effect of perceived father hostility on daughter depressive symptoms is significant when perceived father warmth is at or above 5.

The model testing the father-daughter relationship quality supported the interaction hypothesis. As can be seen in Table 2, perceived father warmth moderated the effects of perceived father hostility for all but one outcome (agency). Each significant interaction was probed and plotted. Figure 2A illustrates the association between perceived father hostility and depressive symptoms at high and low levels of perceived father warmth. Perceived father hostility was associated with higher depressive symptoms (b = 0.53, *p* < 0.05) when perceived father warmth was high (1 SD above the mean), but not when perceived father warmth was low (1SD below the mean). Figure 2B explores the regions of significance for the effects of perceived father hostility on depressive symptoms – perceived father hostility had a significant positive effect on depressive symptoms when perceived father warmth was above 5 - a value that is half a point above the average value of 4.52 for perceived father warmth in our sample.

**Table 2:** Unstandardized and standardized coefficients for the mother-daughter and father-daughter models.

	Model for fathers				Model for mothers				Mother vs. father
	B	SE	C.R.	*β*	B	SE	C.R.	*β*	Δχ^2^ (1)
**Depressive symptoms on**									
Perceived parental warmth (PPH)	−0.19**	0.06	−3.12	−0.38	−0.13*	0.06	−2.19	−0.28	0.47
Perceived parental hostility (PPH)	0.12	0.14	0.88	0.11	0.10	0.11	0.90	0.12	0.01
PPW × PPH	0.32***	0.09	3.41	0.38	0.05	0.07	0.68	0.07	5.96*
**Anxiety symptoms on**									
Perceived parental warmth (PPH)	−1.37	0.73	−1.88	−0.25	−1.36*	0.69	−1.97	−0.26	0.00
Perceived parental hostility (PPH)	1.21	1.68	0.72	0.10	−0.37	1.32	−0.28	−0.04	0.55
PPW × PPH	2.96*	1.14	2.59	0.31	−0.14	0.84	−0.16	−0.02	5.38*
**Romantic partner hostility on**									
Age	0.05	0.02	1.90	0.25	0.04	0.02	1.71	0.22	0.04
Perceived parental warmth (PPH)	0.00	0.05	−0.02	0.00	−0.09*	0.05	−1.92	−0.33	1.79
Perceived parental hostility (PPH)	0.24*	0.12	2.04	0.38	0.01	0.09	0.14	0.02	2.34
PPW × PPH	0.18**	0.07	2.64	0.37	0.10	0.06	1.68	0.23	0.99
**Romantic partner warmth on**									
Age	−0.01	0.03	−0.43	−0.05	−0.07*	0.03	−2.27	−0.27	1.63
Perceived parental warmth (PPH)	0.21**	0.07	3.12	0.48	0.20**	0.06	3.11	0.50	0.01
Perceived parental hostility (PPH)	0.05	0.15	0.34	0.05	0.19	0.12	1.64	0.25	0.51
PPW × PPH	−0.36***	0.08	−4.57	−0.48	−0.14	0.08	−1.75	−0.22	4.84*
**Agency on**									
Perceived parental warmth (PPH)	0.00	0.10	0.02	0.00	0.12	0.09	1.39	0.19	0.86
Perceived parental hostility (PPH)	0.09	0.22	0.41	0.06	−0.30*	0.15	−2.00	−0.32	2.20

* *p* < 0.05;

** *p* < 0.01;

*** *p* < 0.001.

**Figure 3:**
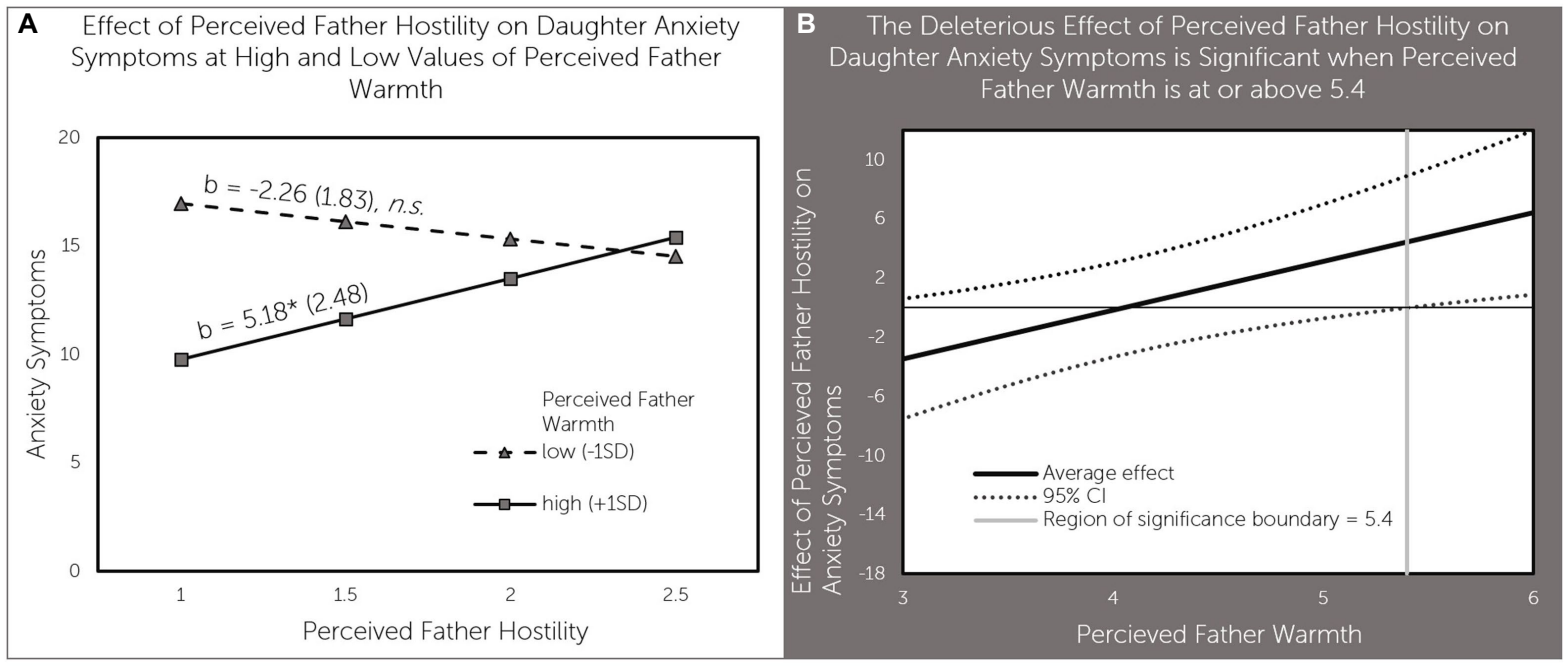
Perceived father warmth moderates the effect of perceived father hostility on daughter anxiety symptoms **(A)** effect of perceived father hostility on daughter anxiety symptoms at high and low values of perceived father warmth; **(B)** The deleterious effect of perceived father hostility on daughter anxiety symptoms is significant when perceived father warmth is at or above 5.4.

Perceived father warmth moderated the association between perceived father hostility and anxiety symptoms in a similar fashion. Perceived father hostility was associated with higher anxiety symptoms (b = 5.18, *p* < 0.05) when perceived father warmth was high (1SD above the mean), but not when perceived father warmth was low (1SD below the mean; Figure 3A). As can be seen in Figure 3B, perceived father hostility had a significant positive effect on anxiety symptoms when perceived father hostility was above 5.4 - a value that is nearly one point above the average value of 4.52 for perceived father warmth in our sample.

**Figure 4:**
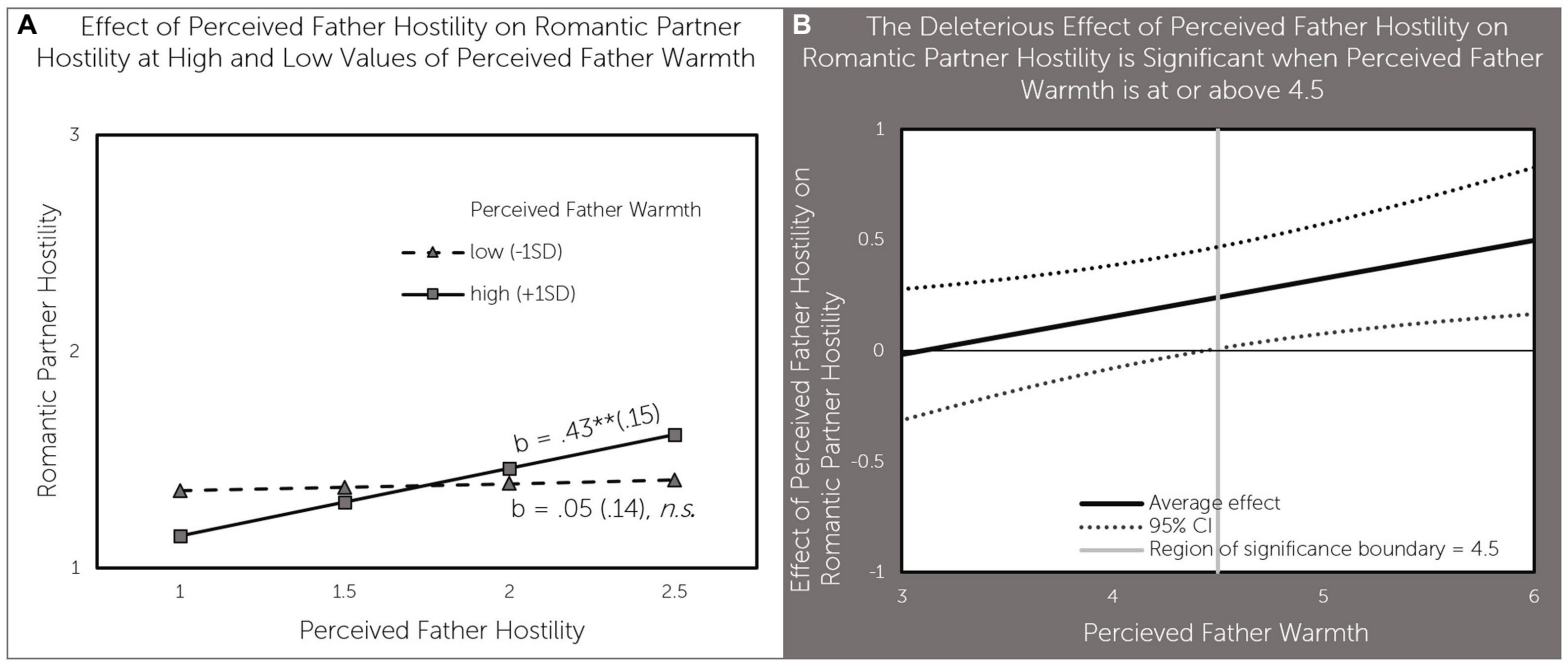
Perceived father warmth moderates the effect of perceived father hostility on daughter’s romantic partner hostility **(A)** effect of perceived father hostility on romantic partner hostility at high and low values of perceived father warmth; **(B)** The deleterious effect of perceived father hostility on romantic partner hostility is significant when perceived father warmth is at or above 4.5.

Perceived father warmth moderated the association between perceived father hostility and romantic partner hostility. Perceived father hostility was associated with higher romantic partner hostility (b = 0.43, *p* < 0.01) when perceived father warmth was high (1SD above the mean), but not when perceived father warmth was low (1SD below the mean; Figure 4A). As can be seen in Figure 4B, perceived father hostility had a significant positive effect on romantic partner hostility when perceived father hostility was above 4.5 - a value that is near the average value of 4.52 for perceived father warmth in our sample.

**Figure 5:**
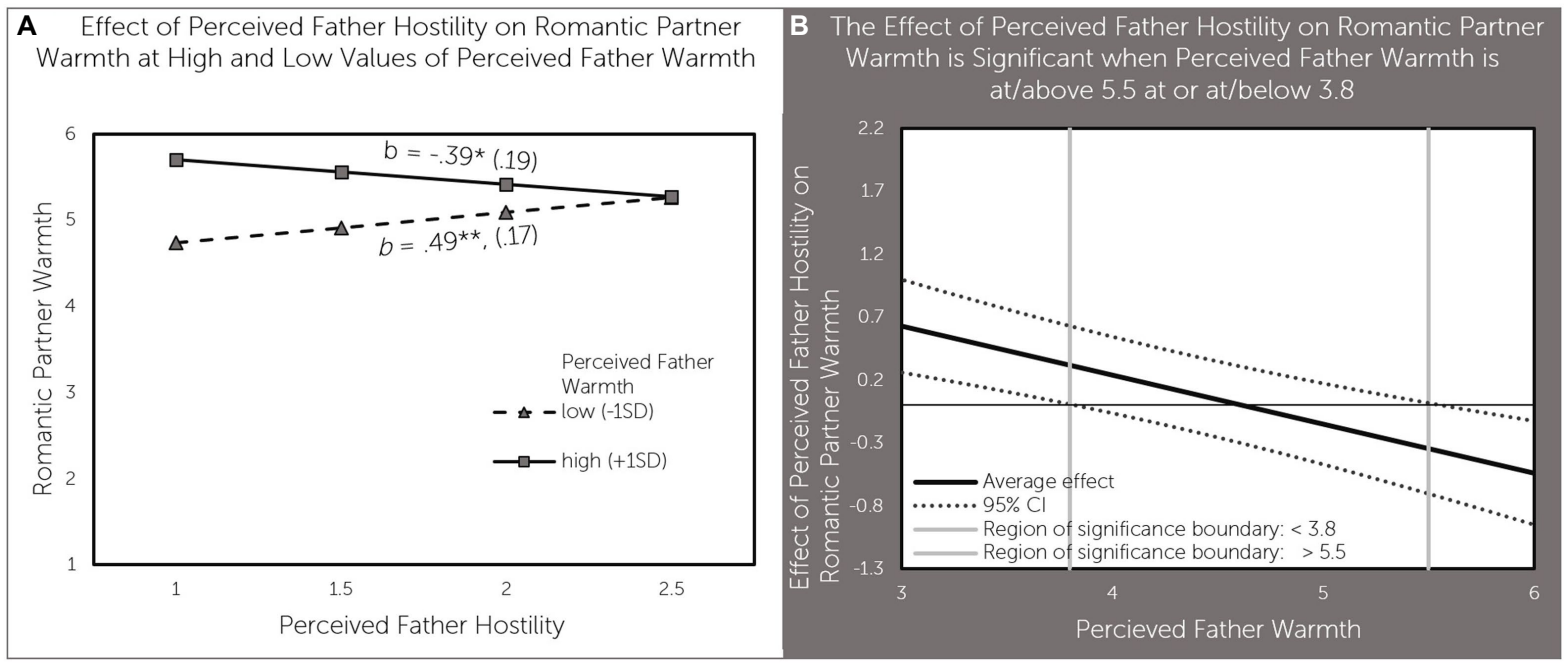
Perceived father warmth moderates the effect of perceived father hostility on daughter’s romantic partner warmth **(A)** effect of perceived father hostility on romantic partner warmth at high and low values of perceived father warmth; **(B)** The deleterious effect of perceived father hostility on romantic partner warmth is significant when perceived father warmth is at or above 5.5; a positive effect of perceived father hostility on romantic partner warmth is significant when perceived father warmth is at or below 3.8.

Finally, perceived father warmth moderated the association between perceived father hostility and romantic partner warmth. Perceived father hostility was associated with lower romantic partner warmth (b = − 0.39, *p* < 0.05) when perceived father warmth was high (1SD above the mean). In contrast, perceived father hostility was associated with higher romantic partner warmth (b = 0.49, *p* < 0.01) when perceived father warmth was low (1SD below the mean; Figure 5A). As can be seen in Figure 5B, perceived father hostility had a significant positive effect on romantic partner warmth when perceived father hostility was below 3.8 and negative effect when perceived father hostility was above 5.5 - a value that is nearly one point above the average value of 4.52 for perceived father warmth in our sample.

Perceived father hostility and warmth were not associated with agency. The model had an excellent fit: χ^2^(4) = 4.08, *p* = 0.39; CFI = 0.99, RMSEA = 0.02. It accounted for 23% of variance in depressive symptoms, 13% of variance in anxiety symptoms, 22% of variance in romantic partner hostility, 34% of variance in romantic partner warmth, and 0.3% of variance in agency.

### 3.4. The mother-daughter relationship model

The model testing the mother-daughter relationship quality did not support the interaction hypothesis (Table 2). Perceived mother warmth was associated with lower depressive symptoms, *β* = −0.28, *p* < 0.05; lower anxiety symptoms, *β* = −0.26, *p* < 0.05; lower romantic partner hostility, *β* = −0.33, *p* < 0.05; higher romantic partner warmth, *β* = 0.50, *p* < 0.01. Perceived maternal hostility was associated with lower agency, *β* = −0.32, *p* < 0.05. The model had an excellent fit: χ^2^(4) = 5.54, *p* = 0.24; CFI = 0.98, RMSEA = 0.07. It accounted for 11% of variance in depressive symptoms, 6% of variance in anxiety symptoms, 13% of variance in romantic partner hostility, 19% of variance in romantic partner warmth, and 6% of variance in agency.

## 4. Discussion

The results of this study highlight the importance of studying father-daughter relationships and mother-daughter relationships separately. Perceived maternal warmth did not moderate the effects of perceived maternal hostility, suggesting the importance of maternal warmth regardless of maternal hostility levels. The importance of the mother-daughter relationship was further supported by our finding that perceived maternal hostility, and not perceived paternal hostility, was associated with lower daughter agency. Although the model explained only a modest amount of variance in agency (6%), this result stands out against the non-significant associations found for the father-daughter model.

Our hypothesis that father-daughter relationship would in general have a stronger association with daughter outcomes was also supported. Comparisons of the mother vs. father interaction coefficients show a significant difference (as indicated by the significant delta child-square test). Furthermore, the model for the father accounted for more variance for nearly all outcomes (with the exception of agency). The pattern of associations of the father-daughter relationship quality with daughter outcomes was, however, surprising. We hypothesized that the co-occurrence of high paternal hostility and low perceived father warmth would result in a synergistic effect associated with the most pronounced deleterious outcomes, whereas high paternal warmth would protect daughters from the negative effects of paternal hostility. In contrast, higher perceived father warmth exacerbated the negative effects of father hostility for daughter internalizing symptoms and romantic relationship quality. Our interpretation of these results must address two separate questions – (1) how might daughters come to view their relationship as both high on perceived warmth and perceived hostility and (2) why would this combination of perceived relationship quality be particularly harmful?

### 4.1. How might daughters come to view their fathers as both warm and hostile?

One of the reasons for fathers to be rated as both warm and hostile is rooted in the self-report nature of the perceived parental warmth and perceived parental hostility measures. Although we observed a high negative correlation between perceived father warmth and perceived father hostility (*r* = −0.50), this correlation may be particularly driven by extreme scores for cases where father warmth was rated as extremely low and father hostility as extremely high. Using a median split, a fair share (20%) of our participants rated their fathers as both warmth and hostile. In cross-cultural research, the co-occurrence of high warmth and high hostility has been hypothesized to occur due to cultural differences in the perceptions of parenting behaviors (Spencer and Swanson, 2013; Zheng et al., 2017). If adolescents perceived hostile behavior as normative, parental hostility may not preclude them from evaluating their parents as warm and supportive. Although we do not have national norms for our parental hostility measure, there is some evidence that our sample was exposed to particularly high levels of hostility within their father-daughter relationship. For example, 32% of them reported their father throwing things at them, 23% reported being threatened with physical violence, 27% were pushed, grabbed, or hit by their father. In comparison, The National Survey of Children’s Exposure to Violence conducted in 2011 (Finkelhor et al., 2013), around the time of this study, estimates that 13.7% of adolescents experienced maltreatment by a caregiver, and 3.7% experiencing physical abuse. Thus, it is possible that the perceived father warmth in our study served as an indicator of closeness in the father-daughter relationship, even when such relationship was not particularly nurturing and supportive.

### 4.2. Why is the combination of high father warmth and high father hostility particularly harmful?

Youth who continue to perceive their father as warm and supportive in the face of high levels of hostility may internalize dysfunctional relationship schemas that have negative consequences for their developmental outcomes. In other words, our findings may not be reflecting that a presence of an objectively warm and supportive father is a risk factor. Instead, the daughter’s perception of her father as warm and supportive is deleterious for daughters growing up with hostile fathers. Furthermore, some hostile parents may not be consistently cold and hostile, but rather intersperse their hostility with bouts of displayed warmth and support (Maccoby and Martin, 1983; Simons and Conger, 2007). Extant literature supports the idea that rejection by a significant person is particularly powerful (Khaleque and Rohner, 2012).

The lessons that the child learns from parental hostility differ depending on the context of this hostility. Straus and colleagues (Straus et al., 1990) have proposed that harsh parenting teaches youth that violence is usual and acceptable. When these lessons occur in the context of intermittent support and positive regard, the child may internalize those lessons that much stronger. In contrast, the parent–child relationship that is consistently cold, rejecting, and hostile may not serve as a model of close relationships (Simons et al., 2012). In the context of our findings, these lessons are not limited to romantic relationships, but also suggest that daughters of hostile/warm fathers internalize the image of self that is expressed in higher depressive and anxiety symptomatology.

Another reason for our observed pattern of effects comes from the social learning theory. A number of studies have shown that imitation is most likely to occur when the observer likes and identifies with the object of their observations (Bandura, 1986). Furthermore, parents high on warmth are more likely to be imitated by their children than parents low on warmth (Hetherington and Frankie, 1967). Thus, daughters may identify and imitate fathers they perceived as warm, in turn being more strongly influenced by their fathers’ hostility.

Consistent with this reasoning Simons et al. (2012) found that high interparental warmth exacerbated the effects of interparental hostility on the college students’ intimate partner violence (IPV) perpetration for both sons and daughters, whereas paternal and maternal warmth and hostility toward their child interacted in their effects on IPV for daughters only. Specifically, father warmth (but not mother warmth) exacerbated the effects of father hostility on daughter perpetration of IPV and both father and mother warmth exacerbated the effects of parental hostility on daughter IPV victimization. In another study (Yoon et al., 2018), results show the effect of paternal abuse on early adolescents’ internalizing and externalizing problems when father involvement is high, and no such effect when father involvement is low.

Finally, exposure to hostility within a close relationship may be particularly damaging to youth mental health. Our finding that paternal hostility is associated with higher depressive and anxiety symptoms only among daughters who view their fathers as warm suggests that greater relationship closeness serves as the vulnerability to negative effects of paternal hostility. In a similar vein, Lansford and colleagues found that spending more time and doing activities with physically abusive fathers is associated with increased internalizing symptoms (Lansford et al., 2002) and the deleterious effect of corporal punishment on anxiety is the highest for children whose mothers are rated as warm (Lansford et al., 2014).

### 4.3. Limitations and future directions

This study relied on data from a single reporter, potentially bolstering the associations between the variables. As such, the associations between perceived warmth variables across multiple relationships may be in part a reflection true overlap across these constructs and to some extent be caused by the participant reporting bias. Past research suggests that the child’s perspective, rather than parents’ perspective, of her relationship with parents is more predictive of the impact that the relationship will have on her psychosocial wellbeing (Shek, 1989; Marsiglio et al., 2000; Rohner and Veneziano, 2001; Khaleque and Rohner, 2002; Beckert et al., 2006). However, it is possible that some participants underreported the extent of problems in their relationships, as well as their depressive and anxiety symptoms, conflating our results with this reporting bias. Future studies corroborating our findings using both participant observations, clinical ratings, and youth self-report would strengthen our understanding of the mechanisms involved in the intergenerational transmission of relationship hostility.

Given the delinquency focus in our sample, our results may not generalize to all adolescents. As such, our participants might have been exposed to more violence (within and outside of their parent–child relationship) and therefore be more inclined to view paternal violent behavior as normative. Furthermore, about half of the participants were incarcerated at the time of the interview. Although their time spent in incarceration had been relatively short at that point (between 1 and 6 months) their reports of relationship quality might have been skewed due to a simple passage of time or due to their idealization of life on the outside. However, an examination of the mean differences between the incarcerated and probation samples on all study variables revealed that these two groups did not differ on the key study variables. Not surprisingly, incarcerated daughters had significantly higher rates of delinquent behavior than daughters in the probation sample (mean = 0.45 for incarcerated girls vs. mean = 0.23 for girls on probation, *t*[88] = 4.90, *p* < 0.001).

Additional limitations stem from the small size of our sample. Given the sample size, we were not able to test the three-way parental hostility x parental warmth x parent gender interaction. Furthermore, we were not able to take advantage of our diverse sample and include a meaningful investigation of the role of race/ethnicity and participant sexual orientation in our findings. Our sample was also limited in focusing on cis-gender heterosexual parents. It is likely that the specific patterns of paternal vs. maternal effects observed in our study are limited to cis-gender heterosexual partnerships; whereas future studies would expand our understanding of the role of parental gender identity and sexual orientation on parenting and parental socialization.

Finally, the study employed cross-sectional data, limiting our ability to make assertions about the directionality of the effects observed in the model. Although the establishment of the father-daughter relationship quality temporally precedes the establishment of the romantic relationship quality, it is also possible that current perceptions of the romantic relationship quality influence daughters’ perceptions of their father-daughter relationship. Most importantly, although we hypothesize that father hostility leads to deleterious daughter outcomes, it is also possible that daughter internalizing and anxiety symptoms influence her reports of father-daughter and romantic relationship quality. Future studies employing longitudinal data will need to test the directionality and significance of these effects.

In conclusion, this study provides a first step in the effort to expand our understanding of the role of father-daughter relationship quality on daughter romantic relationship quality and daughter psychosocial outcomes. It underscores the importance of social relationships in explaining adolescent girls’ psychosocial outcomes. Furthermore, the study confirms the findings of Simons et al. (2012) by demonstrating that high father-daughter relationship warmth does not serve a buffering function, but rather promotes similarities (for better or for worse) between hostility levels in the father-daughter and romantic relationships. These findings have important theoretical implications for the study of parenting and adolescent-father relationships, as well as potential implications for interventions for delinquent youth. As such, interventionists working with delinquent girls and their families may need to differentially focus on parental-adolescent relationships, depending on the amount of hostility within the family context. When family hostility is low, bolstering the sense of warmth and support may be an appropriate target of intervention. However, for youth surrounded by paternal hostility, a better approach might include a focus on learning to identify behaviors that do and do not belong to a healthy intimate relationship.

## Data availability statement

The datasets presented in this article are not readily available because Per IRB human subjects protocol, this study data include restricted access information and cannot be made available for public use. Requests to access the datasets should be directed to JD, julia.dmitrieva@du.edu.

## Ethics statement

The studies involving human participants were reviewed and approved by University of Denver Institutional Review Board. Written informed consent to participate in this study was provided by the participants’ legal guardian/next of kin.

## Author contributions

JD participated in the study design, data collection, data analysis, and manuscript preparation. EE participated in the study design, data collection, and manuscript preparation. All authors contributed to the article and approved the submitted version.

## Conflict of interest

The authors declare that the research was conducted in the absence of any commercial or financial relationships that could be construed as a potential conflict of interest.

## Publisher’s note

All claims expressed in this article are solely those of the authors and do not necessarily represent those of their affiliated organizations, or those of the publisher, the editors and the reviewers. Any product that may be evaluated in this article, or claim that may be made by its manufacturer, is not guaranteed or endorsed by the publisher.
